# Humoral and T-Cell-Mediated Immunity Against *Phlebotomus perniciosus* Salivary Proteins in Dogs from a Leishmaniosis-Endemic Area

**DOI:** 10.3390/pathogens14060576

**Published:** 2025-06-10

**Authors:** Núria Balsells-Aguilar, Maria Magdalena Alcover, Marta Baxarias, Alejandra Álvarez-Fernández, Lourdes Alarcón, Petra Sumova, Petr Volf, Laia Solano-Gallego

**Affiliations:** 1 Departament de Medicina i Cirurgia Animals, Facultat de Veterinària, Universitat Autònoma de Barcelona, 08193 Bellaterra, Spainmartabaxcan@gmail.com (M.B.); alejalfe@gmail.com (A.Á.-F.); lourdes.alarcon@uab.cat (L.A.); 2 Secció de Parasitologia, Departament de Biologia, Salut i Medi Ambient, Facultat de Farmàcia i Ciències de l’Alimentació, Universitat de Barcelona, 08028 Barcelona, Spain; 3 Department of Parasitology, Faculty of Science, Charles University, 12800 Prague, Czech Republic; sumovapetra@seznam.cz (P.S.); volf@cesnet.cz (P.V.)

**Keywords:** anti-saliva antibodies, canine, *Leishmania infantum*, recombinant salivary proteins, specific *P. perniciosus* saliva IFN-γ

## Abstract

Compounds in sand fly saliva elicit specific immune responses that may play a role in the establishment of canine *Leishmania* infection. Although canine antibodies to anti-sand fly saliva antigens have been extensively studied, little is known about cellular immune responses against *Phlebotomus perniciosus* salivary proteins. This study aimed to explore humoral and T-cell-mediated immunity against *P. perniciosus* salivary proteins in dogs (n = 85) from Mallorca (Spain), a leishmaniosis-endemic area, and find correlations with demographic (age, sex, and breed) and parasite-specific immunological parameters. Anti-sand fly saliva IgG was examined using a *P. perniciosus* whole salivary gland homogenate (SGH) ELISA and recombinant salivary protein rSP03B ELISA. Interferon gamma (IFN-γ) release whole blood assays with *L. infantum* soluble antigen (LSA), SGH, and rSP03B were also performed. Positive correlations were found between IgG levels in the SGH and rSP03B tests and between concentrations of SGH IFN-γ and rSP03B IFN-γ. While concentrations of SGH IFN-γ and rSP03B IFN-γ were low and produced only by a minority of dogs (less than 20%), high levels and frequencies of LSA IFN-γ as well as anti-saliva IgG for SGH and rSP03B were detected in a majority of dogs (61% and 75%, respectively). LSA IFN-γ levels were positively correlated with age and *Leishmania*-specific antibodies. In conclusion, dogs from a leishmaniosis-endemic area presented high humoral immunity against *P. perniciosus* salivary proteins, but their cellular immunity to these proteins was low and less frequent.

## 1. Introduction

The leishmanioses are a group of neglected diseases caused by protozoa of the genus *Leishmania* (class Kinetoplasta, family Trypanosomatidae), which are endemic in large areas across the world. There are around 53 known species of *Leishmania*, 31 of which parasitize mammals, and 20 are known to cause disease in humans [1,2]. *Leishmania* is usually transmitted to mammalian hosts by infected female phlebotomine sand flies of the genera *Phlebotomus* in the Old World and *Lutzomyia* in the New World [3]. Several *Phlebotomus* spp. vectors have been identified in the Mediterranean Basin [4], with *P. perniciosus* being predominant in Spain [5,6].

Infection occurs when an infected sand fly female inoculates the host with metacyclic promastigotes while blood feeding. Sand fly saliva contains molecules with a range of pharmacological and immunomodulatory properties that facilitate blood meal, interfere with the immune response, and help establish infection [7]. Saliva from *Phlebotomus* sand flies inhibits lymphocyte proliferation; suppresses early production of interleukin 2 (IL-2), interleukin 4 (IL-4), and interferon gamma (IFN-γ); increases interleukin 6 (IL-6) production; and induces positive macrophage chemotaxis. Moreover, it alters nitric oxide (NO) synthesis in macrophages by inducing down-regulation of NO synthase [8]. Other influential factors include the virulence of the parasite strain and the genetic background and immune status of the host [7].

Canine leishmaniosis caused by *Leishmania infantum* has an almost global distribution and is reported in regions of Southern Europe, Africa, Asia, South and Central America, and the United States of America [9]. Its prevalence is particularly high in the Mediterranean Basin [10] and South America [3]. Infection is expected to grow in non-endemic areas due to climate change, which will create more favorable conditions for both vectors and parasites; additionally, imported cases are becoming more frequent due to increased population mobility and relocation [11].

As in humans, the levels of susceptibility and resistance to leishmaniosis in dogs depend on the immune response, as well as on the complex interactions between host and parasite [12]. Clinical manifestations and disease progression can, therefore, be highly variable. Infection in some individuals may be subclinical or unapparent, while others may show a broad spectrum of clinical signs and/or clinicopathological abnormalities, ranging from cutaneous lesions to severe systemic disease [9].

Some dog breeds are reported to be more susceptible to systemic leishmaniosis, such as the Boxer, Cocker Spaniel, Rottweiler, and German Shepherd [8,12]. The Ibizan Hound seems to be resistant to *Leishmania* infection due to a significant parasite-specific T-cell immune response [13,14]. Compared to other dog breeds, they have a higher tendency to develop papular dermatitis instead of systemic disease [15].

Canine resistance or susceptibility to developing leishmaniosis depends on several factors, including age, sex, immunosuppression, the presence of concomitant diseases or ongoing coinfections, nutritional status, and the amount of time spent outdoors [16]. Genetic components related to breed also play a role in the disease outcome, as many loci are accountable for its progression [17]. Resistance to leishmaniosis, as typically seen in Ibizan Hounds, is associated with the development of a strong Th1 response [16] characterized by the production of proinflammatory cytokines such as IFN-γ. In contrast, more susceptible hosts tend to have a stronger Th2 response, with enhanced production of type 2 cytokines such as IL-10, leading to selective inactivation of some macrophage functions and an increased humoral response, which is unable to control the infection [16].

In canine leishmaniosis-endemic areas, there is evidence that hosts repeatedly exposed to sand fly bites develop anti-saliva antibodies, mostly IgG [7,18,19]. Antibody levels fluctuate with the seasonal variation in sand fly activity and abundance [18]. Older dogs usually have stronger anti-saliva humoral immunity than younger dogs due to their having experienced more sand fly seasons and, therefore, having had higher exposure to sand flies [15,18]. In the case of Ibizan Hounds, their large, upright, and hairless ear pinnae are particularly vulnerable to sand fly bites. One of the hypotheses of this study was that Ibizan Hounds would have higher levels of anti-*P. perniciosus* saliva IgG antibodies and a stronger cellular immune response than other breeds, potentially due to greater exposure to sand fly bites. Studies have found that the parasite-specific immune response in this breed is predominantly cellular, with greater IFN-γ production and higher levels of anti-*P. perniciosus* saliva antibodies [15]. This could account for the aforementioned resistance of the Ibizan Hound to leishmaniosis [15]. However, few studies to date have investigated the relationship between humoral and cellular immune responses to sand fly bites in dogs.

The specific immune responses elicited by compounds in sand fly saliva play a significant role in *Leishmania* establishment and disease outcome [7]. To the best of our knowledge, the canine cellular immune response against *P. perniciosus* salivary proteins has not been previously investigated in the leishmaniosis-endemic area. The aim of this study was, therefore, to explore the humoral and T-cell-mediated immunity against *P. perniciosus* salivary proteins in Ibizan Hounds and dogs of other breeds living on the island of Mallorca (Spain) and to correlate anti-saliva immune responses with demographic parameters (age, sex, and breed) and parasite-specific immunological parameters.

## 2. Materials and Methods

### 2.1. Data of Ibizan Hounds and Dogs of Other Breeds

The dogs enrolled in this study were from Mallorca, the largest of the Balearic Islands, an archipelago in the western Mediterranean Sea, and an endemic area for canine leishmaniosis. According to recent studies, seroprevalence for canine leishmaniosis is around 10% in Spain but as high as 57% in the Balearic Islands [5]. Due to the latitude and climate, *Phlebotomus* sand flies are active in the islands for several months each year, when they act as vectors of leishmaniosis for dogs [6].

In the present study, samples were taken in early September 2019 from 85 owned dogs, 51 of which were Ibizan Hounds and 34 of which were of other breeds. Seventeen crossbreed dogs were included, mainly derived from crosses between hunting and working dogs. The majority of dogs were hunting, shepherd, or guardian dogs, and the dogs were born on Mallorca Island, and they did not have travel history. All the studied dogs resided in the same area and shared the same environmental conditions. All lived outdoors and were therefore exposed to sand fly bites. The dogs were not routinely treated with external anti-parasite treatments containing sand fly repellents.

Basic clinical characteristics were recorded: breed, sex (male n = 24, female n = 60, one dog with sex not recorded), and age (from 4 months to 12 years). The median age of the entire sample of dogs was 24 months, with 50/85 (59.5%) above 18 months of age and qualified as adults in this study. The Ibizan Hound group had a lower median age and a lower proportion of adult dogs (median: 18 months; 23/50 or 46% adults, *p* = 0.0018, Fisher’s exact test) than the group of other breeds (median: 36 months; 27/34 or 79.4% adults). Regarding sex, the Ibizan Hounds were predominantly female (43/50 or 86%), whereas the mixed group was equally proportioned between male and female (17/34 or 50%). Clinical status was assessed by veterinarians based on clinical history and physical examination. The majority of dogs were apparently healthy. Only four dogs presented typical cutaneous clinical signs compatible with leishmaniosis [20]. Two young (12 months of age) Ibizan Hounds presented papular dermatitis [15] in the pinna and were low seropositive against *L. infantum* antigen. Two dogs showed medium antibody levels against *L. infantum* antigen and presented exfoliative dermatitis and dermatitis in bony prominences.

Clinical examination and *L. infantum*-specific antibody detection were evaluated as annual screening by veterinarians. Blood samples were taken to obtain sera for serology. Residual blood was used for propagating primary whole blood cultures and determining anti-*P. perniciosus* IgG. A signed consent form was obtained from all the owners of the enrolled dogs.

### 2.2. Cytokine Release Whole Blood Assay

After taking blood samples from both groups of dogs, heparinized whole blood was diluted to a ratio of 1:10 using Rosewell Park Memorial Institute (RPMI) 1640 medium with stable glutamine and 25 mM hepes (Biowest, Nuaill, France) supplemented with penicillin and streptomycin (Life Technologies^TM^, Carlsbad, CA, USA) and 10% Fetal Bovine Serum Premium South America Origin (Quimigen S.L., San Sebastián de los Reyes, Spain). A total of 50 µL of heparinized blood was mixed with 450 µL of complete medium in each well.

Five different conditions were established to stimulate the whole blood:

(i) Medium alone;
(ii) Medium with *L. infantum* soluble antigen (LSA) at a concentration of 10 µg/mL;
(iii) Medium with mitogen concanavalin A (ConA) (100 mg, Medicago, Uppsala, Sweden) at a concentration of 10 µg/mL;
(iv) Medium with *P. perniciosus* salivary gland homogenate (SGH) at a concentration of 1 salivary gland/mL;
(v) Medium with 43-kDa yellow-related recombinant protein (rSP03B) from salivary gland *P. perniciosus* at a concentration of 20 µg/mL.

The preparations were incubated in 48-well flat bottom plastic culture plates 30048 (SPL Life Sciences Co., Ltd., Pocheon-si, Republic of Korea) for 5 days at 37 °C in 5% of CO_2_ air. After 5 days of incubation, the blood was centrifuged at 323× *g* for 10 min, and the supernatant was collected and stored at −80 °C until the IFN-γ analysis.

### 2.3. Sandwich ELISAs to Determine IFN-γ Concentration

After the whole blood assay, an optimized indirect enzyme-linked immunosorbent assay (ELISA) was carried out to measure production of IFN-γ, according to the manufacturer’s instructions (DuoSet ELISA by-R&D Systems, Minneapolis, MN, USA) with slight modifications, using 96-well cell flat bottom plates (ref. 3590, Costar^®^ Corning, Corning, NY, USA). The standard curve for IFN-γ started with 2000 pg/mL, and two-fold dilutions were made until a concentration of 62.5 pg/mL was reached. Supernatants treated with ConA were diluted 1:1 with reagent diluents. All the supernatants were studied in duplicate. Optical density was measured with an ELISA reader (MB-580, Shenzhen Heales Technology Development Co., Ltd., Shenzhen, China) at a wavelength of 450 nm.

The standard curve for IFN-γ was calculated using a computer-generated four-parameter logistic curve fit with the program MyAssays: http://www.myassays.com/ (accessed 30 March 2025). Plates were repeated when the r^2^ value of the standard curve was below 0.98.

Also, dogs were classified as IFN-γ producers with LSA, SGH, and rSP03B antigens when the IFN-γ concentration was detectable after subtracting the medium or as IFN-γ non-producers when concentrations were not at detectable levels (<62.5 pg/mL) [21].

### 2.4. ELISA to Determine Anti-L. infantum Antibodies

All dogs were serologically tested by a quantitative in-house ELISA to measure specific antibodies against *L. infantum*, as previously described [22]. Additionally, 96-well ELISA Costar^®^ plates (Corning, Corning, NY, USA) were coated with 100 µL of sonicated *L*. *infantum* promastigotes at a concentration of 20 µg/mL in 0.05M carbonate buffer, pH 9.6, and then incubated at 4 °C overnight. A total of 100 µL of canine sera was added at a 1/800 dilution in phosphate saline buffer (PBS, pH 7.4) (Sigma-aldrich, Darmstadt, Germany), with 0.05% Tween 20 (PBTS) and 1% low-fat dry milk (PBTSL), and incubated at 37 °C for 60 min. High positive and negative controls, as well as positive calibrator and conjugate controls, were added to each plate. After washing the plates three times with PBST, 100 µL of Protein A peroxidase (Thermo Fisher Scientific, Waltham, MA, USA, dilution 1:30,000) conjugate was added using a 0.16 ng/mL concentration and incubated at 37 °C for 60 min. After further washing, carried out as before, the solution was developed using 200 µL of substrate solution, 0.4 mg/mL OPD, 0.4 mg/mL urea hydrogen peroxide, and 0.05 M phosphate–citrate, pH 5.0 (Sigma-Aldrich, Darmstadt, Germany) and then incubated for 5–20 min at room temperature. The reaction was stopped by adding 50 µL of H_2_SO_4_ 3M. The reading was carried out with an ELISA reader (MB-580, Shenzhen Heales Technology Development Co., Shenzhen, China) at 492 nm.

The results were quantified as ELISA units (EU) related to the positive calibrator, with 100 EU given an optical density of approximately 1. Cut-off was established at 35 EU by the standard deviation (SD) method, consisting of multiplying the SD of the results by four and adding up the mean of the results obtained by the ELISA results of 80 dogs from a non-endemic area, as previously described [23]. To calculate the results, the EU-obtained problem sera were divided by the calibrator EU and then multiplied by 100. Results ≥300 EU were considered highly positive, ≤150 and <300 EU were medium positive, <150 and >35 EU were low positive, and <35 EU were negative. According to these values, dogs were initially classified as negative (n = 55), low positive (n = 27), and medium positive (n = 3). However, because of the low number of medium positive results, the analysis was finally carried out considering all dogs as either positive or negative.

### 2.5. ELISA to Determine Anti-P. perniciosus IgG

Serum samples were analyzed by an ELISA, in which anti-*P*. *perniciosus* IgG was measured using *P*. *perniciosus* salivary gland homogenate (SGH) as previously described [15] or recombinant protein rSP03B with slight modifications [24]. After reconstitution of *P*. *perniciosus* SGH and lyophilized rSP03B with dH_2_O, 96-well ELISA Costar^®^ plates (Corning, Corning, USA) were coated with *P*. *perniciosus* SGH (0.2 salivary glands per well) or recombinant protein rSP03B (0.1 µg per well) diluted in carbonate–bicarbonate buffer (pH 9, 100 μL per well) and incubated overnight at 4 °C. The plates were washed twice with PBS + 0.05% Tween 20 (PBTS) (100 µL per well) and blocked with 6% low-fat dry milk diluted in PBTS (100 µL per well) for 60 min at 37 °C. After that, the plates were washed three times with PBS-Tw (100 µL per well).

Serum samples were diluted 1:200 (for SGH) and 1:100 (for rSP03B) in 2% low-fat dry milk and added to the plates. Each sample was prepared at least in duplicate, incubated for 90 min at 37 °C, and washed five times with PBTS (100 µL per well). After adding a conjugate (HRP; peroxidase-conjugated goat anti-dog IgG antibody, Bethyl Laboratories, Montgomery, AL, USA; A40-123P), the plated samples were incubated again at 37 °C for 45 min, diluted 1:9000 in PBTS (100 µL per well), and washed five times with PBTS.

The ELISA was developed using a substrate solution (200 µL per well) composed of 0.4 mg/mL OPD, 0.4 mg/mL urea hydrogen peroxide, and 0.05 M phosphate–citrate, pH 5.0 (SIGMAFAST OPD, Sigma-Aldrich, Darmstadt, Germany). The plates were incubated in the dark at room temperature, and the reaction was stopped after 5–20 min with 50 µL 10% H_2_SO_4_ 5N. The absorbance (OD value) was measured at 492 nm using an ELISA reader (MB-580, Shenzhen Heales Technology Development Co., Ltd., Shenzen, China). Each serum was tested in duplicate.

The cut-off value for SGH was established at 38.05 ELISA units (EU) in accordance with a previous study [15]. The cut-off value for rSP03B was calculated using 24 control residual samples obtained from the Royal Veterinary College (London, UK), which were collected from a random set of dogs treated at the Queen Mother Veterinary Teaching Hospital [15], together with nine control residual samples from healthy young adult Beagles from Barcelona always under treatment with sand fly repellents. Therefore, it was assumed that these dogs were unexposed to sandflies. The receiver operating characteristic (ROC) curve was generated using GraphPad Prism 9.1.0 (221) software, and a cut-off value of 40.23 EU was used thereafter. High positive and negative controls, as well as positive calibrator and conjugate controls, were added to each plate.

### 2.6. Statistical Analysis

Data analysis was performed with a combination of R software (version 4.5.x; http://cran.r-project.org/, accessed on 30 March 2025) and Quickcalcs from Graphpad (version 1.x; www.graphpad.com/quickcalcs/, accessed on 30 March 2025). Diagnostic performance was calculated based on the degree of agreement between pairs of tests, using McNemar’s test and Cohen’s kappa coefficient. The interpretation of kappa value is shown according to the following scale: ≤0, no agreement; 0.00–0.20, slight; 0.21–0.40, fair; 0.41–0.60, moderate; 0.61–0.80, substantial; 0.81–1, almost perfect. Correlations between the test results were estimated using Spearman’s rho correlation and linear regression after transforming the EU to logarithmic values to normalize residuals and stabilize the variance. The Shapiro–Wilk test was performed to detect normal distribution of quantitative variables. The Mann–Whitney–Wilcoxon test was used to examine intra-group differences in median EU values between tests and demographic parameters. Proportions of intra-group were compared using Fisher’s exact test. Results were expressed as medians (interquartile range, IQR). *p*-values < 0.05 were considered statistically significant.

## 3. Results

### 3.1. Agreement Between Tests

Pairwise analysis was used to assess the agreement between *L*. *infantum*-specific antibodies, the two salivary antibody tests (SGH IgG, rSP03B IgG), and the three IFN-γ tests (LSA IFN-γ, SGH IFN-γ, and rSP03B IFN-γ) (Table 1). A high level of agreement (78.82%) was observed between SGH IgG and rSP03B IgG (*p* = 0.8137) (κ = 0.554 ± 0.093). The agreement was fair (65.88%) between SGH IFN-γ and rSP03B IFN-γ (*p* = 0.1374) (κ = 0.256 ± 0.105) and between *L*. *infantum*-specific antibodies and SGH IgG (62.35%) (*p* < 0.001) (κ = 0.294 ± 0.088).

### 3.2. Spearman’s Rho Correlation and Linear Regression

A Spearman’s rho correlation was used to identify possible correlations between antibodies against salivary antigens and age and other immunological parameters (Table 2). A significant positive correlation was observed between the *L*. *infantum*-specific antibodies and SGH IgG (*p* = 0.0001) and rSP03B IgG (*p* = 0.0113) and between SGH IgG and rSP03B IgG (log EU). A negative correlation was observed between log rSP03B IFN-γ concentrations and SGH IgG EU (*p* = 0.0361).

When analyzing other parameters, significant positive correlations were observed between *L*. *infantum*-specific antibodies (log EU) and LSA IFN-γ (*p* = 0.0121) and between rSP03B IFN-γ and SGH IFN-γ (log EU) (*p* = 0.0004). Notably, a statistically significant positive correlation was obtained between age and LSA IFN-γ (log EU) (Table 2). A significant positive correlation was also found between age and *L*. *infantum*-specific antibodies (log EU) (r^2^ =0.4116) (*p* = 0.0001).

### 3.3. Proportion of Positive Diagnostic Tests and Median Results for All Dogs Studied

The Ibizan Hounds were compared with the other dog breeds in their antibody response to the two *P*. *perniciosus* salivary antigens and in IFN-γ production after stimulation with LSA, SGH, and rSP03B. The proportion of positive results and the median and interquartile values for the six tests performed are displayed in Table 3 and Table 4. The proportion of positive results for rSP03B IgG differed significantly between Ibizan Hounds (72.54%) and the other dog breeds (44.11%) (Fisher’s exact test: *p* = 0.01229). The rSP03B EU also differed significantly between the two groups, with median values higher for Ibizan Hounds (50.81 EU) than the other breeds (36.56 EU) (Mann–Whitney: *p* = 0.0331). Around 40% of all the dogs produced SGH IFN-γ, and 29.4% produced rSP03B IFN-γ. Moreover, 17.6% (15/85) produced both types of specific sand fly salivary IFN-γ.

Interestingly, statistically significant differences were obtained when comparing the median concentrations of LSA IFN-γ (854.40 pg/mL) with SGH IFN-γ (26.75 pg/mL) (Mann–Whitney: *p* < 0.001) and LSA IFN-γ (854.40 pg/mL) with rSP03B IFN-γ (19.25 pg/mL) (Mann–Whitney: *p* < 0.001). Although differences in the median concentrations of LSA IFN-γ were not statistically significant between groups, values were higher for Ibizan Hounds (1015.59 pg/mL) than the other breeds (445.75 pg/mL).

### 3.4. Relationship Between Demographic Parameters and Parasite-Specific Immunological Parameters, P. perniciosus Salivary Antibodies and IFN-γ Concentrations

No statistically significant differences were observed between the sexes. Age seemed to play a role in LSA IFN-γ production, with young dogs giving much lower median values (388.40 pg/mL) than adults (1381.18 pg/mL) (Mann–Whitney: *p* = 0.0031). The proportion of positive results was also significantly higher in adult dogs (82% vs. 35.3%) (Table 5). Adult Ibizan Hounds had a higher IFN-γ response rate (91.3% vs. 66.6%, Fisher’s Exact Test: *p* = 0.048) and significantly higher IFN-concentrations (median 2016.94 pg/mL vs. 628.74 pg/mL, Mann–Whitney: *p* < 0.001) compared to young Ibizan Hounds. However, seropositivity percentages were similar between age groups in Ibizan Hounds (29.6% in young vs. 34.7% in adults, Fisher’s Exact Test: *p* = 0.77). No differences were observed in the mixed-breed group regarding parasite-specific immunological parameters and age.

A statistical difference in the proportion of positives for SGH IgG was observed according to *L*. *infantum* serological status; 83.3% of dogs positive for *L*. *infantum*-specific antibodies also had raised levels of SGH IgG (Fisher’s exact test: *p* = 0.0023), and *L*. *infantum*-seropositive dogs gave higher SGH IgG median values than negative dogs (54.42 vs. 36.35 EU) (Mann–Whitney: *p* = 0.0029) (Table 6). A clear association was seen between SGH IgG and rSP03B IgG production, as 82.7% of rSP03B IgG-positive dogs were also producing SGH IgG, and vice versa (*p* < 0.001). Differences were observed in the proportion of positives for the SGH ELISA between rSP03B IFN-γ producers (44%) and non-producers (68.3%) (Fisher’s exact test, *p* = 0.0506) (Table 6).

### 3.5. SGH IFN-γ and rSP03B IFN-γ

When calculating median concentration values of SGH IFN-γ and rSP03B IFN-γ and Mann–Whitney *p*-values in relation to all clinical and immunological parameters, only values obtained from producer dogs were included, as non-producers of SGH IFN-γ accounted for 60% (51/85) of all dogs and non-producers of rSP03B IFN-γ for 70.5% (60/85). If not, median concentrations calculated from all dogs would have given values around 0. Subsequently, a significant difference in the proportion of positive results was observed for SGH IFN-γ between the rSP03B IFN-γ producers (60%) and non-producers (31.7%) (Fisher’s exact test, *p* = 0.0275), although median values did not reach statistical significance (Table 7). Similarly, a marked difference in the proportion of positive results was observed for rSP03B IFN-γ between the SGH IgG producers (21.15%) and non-producers (42.42%) (Fisher’s exact test, *p* = 0.0359), although median values again did not reach statistical significance (Table 7). Statistical differences were observed in the median values of rSP03B IFN-γ between SGH IFN-γ producers (35.3 pg/mL) and non-producers (2.62 pg/mL) (Mann–Whitney, *p* = 0.0192), with a significantly higher proportion of positives among the producers (44.1%) than the non-producers (19.6%) (Fisher’s exact test, *p* = 0.0275) (Table 7).

## 4. Discussion

It is well known that repeated exposures to sand fly bites elicit both humoral and cellular adaptive immune responses in rodent models and humans and other mammalian hosts [8,25]. However, more limited information is available regarding cellular immune responses after exposure to sand fly bites when compared with humoral immune responses, and it has been mainly studied in rodent models and humans [8,25]. Moreover, cellular immunity to saliva or distinct salivary proteins protects against leishmaniasis in various animal models [26]. To the best of the knowledge of the authors, only two studies have investigated cellular immune response against sand fly salivary proteins experimentally [27] or in nature [26] in dogs. The present study evaluated, for the first time, cellular immune responses against *P*. *perniciosus* whole salivary proteins and the recombinant yellow protein (rSP03B) in dogs.

In the present study, approximately 30–40% of the dogs exhibited IFN-γ in response to rSP03B or SGH, respectively. However, only 17.6% of the dogs produced both types of IFN-γ specific to sand fly saliva. Furthermore, a lower concentration of IFN-γ was found when the blood culture was stimulated with SGH or rSP03B than with LSA, as previously reported in humans [28]. A similar study carried out in humans explored cellular and humoral responses to *Phlebotomus papatasi* saliva in areas of Tunisia where *Leishmania major* is endemic, although with variable degrees of prevalence [28]. It was concluded that individuals frequently exposed to sand fly bites have a strong anti-saliva IgG response [28]. However, less than 30% of the subjects exhibited lymphocyte proliferation against salivary gland extracts in blood cultures, regardless of immune status or area of residence [28]. In a study conducted in Tbilisi (Georgia), an endemic area for human visceral leishmaniasis, and where sand flies are abundant for a short period of ≤3 months. Only 30% of humans and 50% of dogs displayed an antibody response to saliva after the end of the sand fly season [26]. Furthermore, stimulation with *Phlebotomus kandelakii* SGH canine peripheral blood mononuclear cells did not induce any of the tested cytokines (IFN-γ, IL-10, IL12p40, TNF-α, and IL-6) [26]. The present and previous [26] findings in dogs suggest that the cellular immune response to *Phlebotomus* saliva is variable, which could reflect differences in sand fly exposure (less exposure in dogs from Georgia), differences in immunogenic saliva proteins in different sand fly species, immune status of dogs, or the immune system’s ability to recognize the antigens present in sand fly saliva. Further studies are needed regarding cellular immune responses against sand fly salivary proteins to elucidate the relationship between sand fly exposure and clinical outcomes of *Leishmania* infection in dogs and humans.

Regarding salivary antibody levels, a significant percentage of agreement (78.8%) was found between the SGH IgG and rSP03B IgG results. These results are very similar to those of previous studies from Italy and Portugal [18,19], including the one carried out in Mallorca by Burnham et al. [15], where the level of agreement between the same tests was 75.8%. The composition of SGH, which includes a wide range of salivary proteins in their native form, could be modified by colony-related factors. Therefore, SGH could have antigenic properties and potentially cause weak sera cross-reactions with other sand fly species salivary molecules, as previously documented [7,24,25]. In contrast, recombinant proteins such as rSP03B are likely more specific. Interestingly, the potential cross-reactivity of recombinant SP03B protein was studied by Willen et al. [24] using Western blotting. Authors demonstrated that it does not cross-react with sera of animals (mice or dogs) experimentally exposed to sand flies *P*. *papatasi* and *Phlebotomus sergenti*, as well to other hematophagous arthropods, including *Ctenocephalides felis*, *Ixodes ricinus*, and *Culex molestus* [24].

One of the hypotheses of this study was that Ibizan Hounds would have higher levels of anti-*P*. *perniciosus* saliva IgG antibodies, as well as a higher cellular immune response than dogs of other breeds due to greater exposure to sand fly bites [15]. However, only significant differences were found in Ibizan Hounds when compared with other breeds on rSP03B IgG with higher levels and proportion of positive dogs. These findings might imply a higher frequency of sand fly bites in Ibizan Hounds as previously described [15] since this breed has large upright hairless ears opposite to more pendular and hairy ears of other breed groups studied. Therefore, Ibizan Hounds appear to be more attractive to sand fly bites than other breed dogs. In the present study, it is unlikely that this difference in sand fly biting frequency was due to different environmental conditions between groups because all dogs slept outdoors and received inconsistent insecticide treatment. Interestingly, Burnham et al. [15] reported that the median SGH EU was statistically higher in Ibizan Hounds when compared with other breed dogs but not the rSP03B EU. In the latter study, blood samples were taken in December, and the median values for SGH and rSP03B for all the studied dogs were 25–30 EU, in comparison with 36–50 EU in the present study, where the sampling was carried out in September. This difference appears to confirm that antibody levels against sand fly saliva fluctuate throughout the year according to the abundance of vectors [6,19]. In the island of Mallorca, due to its location in the Mediterranean Sea, sand flies are active for long periods each year. The season is estimated to begin between April and mid-June, with sand fly density usually peaking between July and September and activity ceasing from mid-September to November [6]. The risk of leishmaniosis transmission between December and March is negligible [6]. A study in northwestern Spain also found associations between vector exposure and salivary antibody levels, which were lowest during the winter months [29].

The results indicate a moderate agreement between the *L*. *infantum*-specific antibody test and the results obtained for SGH IgG and rSP03B IgG, with significant correlations for both. Additionally, dogs with *L*. *infantum*-specific antibodies were more likely to test positive for SGH IgG and rSP03B, as well as having higher antibody levels. These findings align with previous studies [19,30] showing that *L*. *infantum-*seropositive dogs also exhibit elevated levels of anti-saliva antibodies. Therefore, the detection of sand fly salivary IgG in dogs is an excellent marker to assess the risk of *L*. *infantum* infection, and it should be implemented as a tool to determine good areas to carry out clinical studies, such as vaccine field trials and to perform vector control campaigns.

Notably, the LSA IFN-γ results also showed 50.5% agreement with the *L*. *infantum*-specific antibody test and 57.6% with the SGH ELISA. A positive Spearman correlation was obtained between log LSA IFN-γ units and the *L*. *infantum*-specific antibody test. Furthermore, a significant correlation was found between age and LSA IFN-γ, with median values increasing in older dogs, as similarly reported in human leishmaniosis [31,32,33]. In addition, a significant correlation was also found between age and *L*. *infantum* antibody levels, as previously described [34,35,36]. Previous studies have reported that a low production of LSA IFN-γ is associated with clinical leishmaniosis and unfavorable clinical outcomes [21].

In both our study and that of Burnham et al. [15], the Ibizan Hounds had a lower median age than the mixed breed group, which influenced the results. Younger dogs tend to have weaker immune responses due to having experienced fewer sand fly seasons [19]. Accordingly, we found a statistical difference in the relationship between LSA IFN-γ and age, with young Ibizan Hound dogs showing much lower median values than adult Ibizan Hound dogs. The proportion of positive results was also significantly higher in the adult Ibizan Hound dogs when compared with young dogs. Mixed breed dogs had the same tendency but were not significant due to the lower number of dogs in this group. Interestingly, seropositivity percentages were similar between age groups in Ibizan Hounds. This suggests that age influences cellular immune responses (IFN-γ production) more than humoral immunity (seropositivity) in this breed. These findings reinforce that age is a key factor in cellular immune competence, particularly in Ibizan Hounds. Age was not found to have a clear influence on the mean concentration values of SGH IFN-γ and rSP03B IFN-γ or the proportion of producing dogs. However, the relatively low percentage of positives in these tests makes it difficult to draw a reliable conclusion. Thus, the number of dogs studied may have been too small to be considered a representative sample.

Many of our results agree with those of previous studies that have established a clear relationship between the humoral immune response to *Leishmania* infection and *P*. *perniciosus* saliva proteins [7,37]. This correlation is because dogs highly exposed to sand flies have a higher chance of becoming infected by *Leishmania*. However, to the best knowledge of the authors, there is so far no clear data to suggest that the immune response to saliva influenced the *Leishmania* immune response in dogs. Unfortunately, we did not observe enough difference between Ibizan Hounds (considered more resistant to developing clinical illness) versus other breeds of dogs [13,14,15]. In addition, there was not a clear relationship between parasites’ and sand flies’ salivary cellular immune responses. The connection found between SGH IFN-γ and rSP03B IFN-γ and the other analyzed parameters calls for further research to explore their role in mechanisms of cellular immunity. This would include comparisons with other markers of immunity and disease. Furthermore, the dogs enrolled in this study were a relatively homogenous sample: all lived in the same area endemic for canine leishmaniosis, and all were exposed to the same environmental conditions and were mainly *Leishmania*-seronegative or low positive. In future studies, it would be of interest to include dogs in different clinical states of infection, ranging from healthy dogs from a non-endemic area to dogs with very severe clinical leishmaniosis, and to collect samples at different times of the year (longitudinal studies).

## 5. Conclusions

In conclusion, dogs from a leishmaniosis-endemic area presented high humoral immunity against *P*. *perniciosus* salivary proteins, but their cellular immunity to these proteins was low and less frequent. The present study corroborates that Ibizan Hounds appear to be more attractive to sand fly bites than other breed dogs since they present higher humoral immunity against *P*. *perniciosus* salivary proteins. However, no differences between breeds were found regarding cellular immunity to these salivary proteins.

## Figures and Tables

**Table 1:** Agreement between *L*. *infantum*-specific antibody test, *P*. *perniciosus* salivary antibody tests, and IFN-γ ELISA results (LSA IFN-γ, SGH IFN-γ, and rSP03B IFN-γ).

Pair Parameters	Percent Agreement	McNemar’s Exact Test *p*-Value	Kappa ± SE	Kappa Interpretation Ϯ
SGH IgG vs. rSP03B IgG	78.82%	0.8137	0.554 ± 0.093	Moderate agreement
SGH IgG vs. LSA IFN-γ	57.65%	0.0668	0.045 ± 0.104	Slight agreement
SGH IgG vs. SGH IFN-γ	41.18%	0.0162 *	−0.126 ± 0.100	No agreement
SGH IgG vs. rSP03B IFN-γ	35.29%	0.0005 ***	−0.185 ± 0.092	No agreement
rSP03B IgG vs. LSA IFN-γ	55.29%	0.0744	−0.008 ± 0.103	No agreement
rSP03B IgG vs. SGH IFN-γ	45.88%	0.0122 *	−0.036 ± 0.099	No agreement
rSP03B IgG vs. rSP03B IFN-γ	37.65%	0.0004 ***	−0.142 ± 0.091	No agreement
LSA IFN-γ vs. SGH IFN-γ	41.18%	<0.0001 ***	−0.068 ± 0.085	No agreement
LSA IFN-γ vs. rSP03B IFN-γ	44.71%	<0.0001 ***	0.085 ± 0.066	Slight agreement
SGH IFN-γ vs. rSP03B IFN-γ	65.88%	0.1374	0.256 ± 0.105	Fair agreement
*L*. *infantum-*specific antibodies vs. SGH IgG	62.35%	0.0002 ***	0.294 ± 0.088	Fair agreement
*L*. *infantum-*specific antibodies vs. rSP03B IgG	55.29%	0.0007 ***	0.061 ± 0.092	Slight agreement
*L*. *infantum-*specific antibodies vs. LSA IFN-γ	50.59%	0.0001 ***	0.140 ± 0.073	Slight agreement
*L*. *infantum*-specific antibodies vs. SGH IFN-γ	50.59%	0.6434	−0.050 ± 0.107	No agreement
*L*. *infantum-*specific antibodies vs. rSP03B IFN-γ	61.18%	0.4862	0.117 ± 0.110	Slight agreement

Ϯ the interpretation of Cohen’s kappa value is shown in the final column according to the following scale: ≤0, no agreement; 0.00–0.20, slight; 0.21–0.40, fair; 0.41–0.60, moderate; 0.61–0.80, substantial; 0.81–1, almost perfect. Significant *p-*values are designated with an asterisk (* *p* < 0.05, *** *p* < 0.001). Abbreviations: ELISA, enzyme-linked immunosorbent assay; IFN-γ, interferon gamma; LSA, *L*. *infantum* soluble antigen; rSP03B, 43-kDa yellow-related recombinant protein; SE, standard error; SGH, salivary gland homogenate; vs., versus.

**Table 2:** Relationship between log salivary antigen ELISA units and log IFN-γ concentration with other quantitative clinical and immunological parameters using Spearman’s rho correlation and linear regression.

Age and Immunological Parameters	Log Salivary Antigen Units				Age and Immunological Parameters	Log IFN-γ Concentration					
	SGH ELISA		rSP03B ELISA			LSA IFN-γ		SGH IFN-γ		rSP03B IFN-γ	
	r_2_	*p*-Value	r_2_	*p*-Value		r_2_	*p*-Value	r_2_	*p*-Value	r_2_	*p*-Value
Log *L*. *infantum-*specific antibodies ELISA units	0.4077	0.0001 ***	0.2742	0.0113 *	Log *L*. *infantum-*specific antibodies ELISA units	0.2708	0.0121 *	−0.1446	0.1866	0.0124	0.9098
Log LSA IFN-γ units	0.1654	0.1302	0.0938	0.3928	Log SGH IFN-γ units	0.0215	0.8445				
Log SGH IFN-γ units	−0.0418	0.7035	0.0679	0.5363	Log rSP03B IFN-γ units	0.0732	0.5051				
Log rSP03B IFN-γ units	−0.2276	0.0361 *	−0.1515	0.1661	Log rSP03B IFN-γ units			0.3740	0.0004 ***		
Age (months)	−0.0326	0.7682	-0.0939	0.3951	Age (months)	0.2726	0.0121 *	0.0231	0.8346	0.0765	0.4887
Log rSP03B salivary antigen ELISA units	0.7024	<0.0001 ***									

Log_10_ transformations were performed for each variable involving ELISA units (salivary antigens, *L*. *infantum* antigen) and IFN-γ concentration. Significant *p-*values are designated with an asterisk (* *p* < 0.05, *** *p* < 0.001). Abbreviations: ELISA, enzyme-linked immunosorbent assay; IFN-γ, interferon gamma; LSA, *L*. *infantum* soluble antigen; rSP03B, 43-kDa yellow-related recombinant protein; SGH, salivary gland homogenate.

**Table 3:** Proportion of positive dogs for any of the diagnostic tests used.

Test	Total Proportion of Positives (n = 85)	Proportion of Positive Ibizan Hounds (n = 51)	Proportion of Positive Other Breeds (n = 34)	Fisher’s Exact Test *p-Value*
*L*. *infantum-*specific antibodies	35.29% (30/85)	31.37% (16/51)	41.17% (14/34)	0.3659
SGH IgG	61.17% (52/85)	62.74% (32/51)	58.82% (20/34)	0.8211
rSP03B IgG	61.17% (52/85)	72.54% (37/51)	44.11% (15/34)	0.01229 *
LSA IFN-γ	75.29% (64/85)	78.40% (40/51)	70.60% (24/34)	0.4494
SGH IFN-γ	40% (34/85)	35.29% (18/51)	47.05% (16/34)	0.3666
rSP03B IFN-γ	29.41% (25/85)	27.45% (14/51)	32.35% (11/34)	0.6360

Abbreviations: IFN-γ, interferon gamma; LSA, *L*. *infantum* soluble antigen; rSP03B, 43-kDa yellow-related recombinant protein; SGH, salivary gland homogenate. Fisher’s exact test was conducted as a two-tailed analysis. Significant *p-*values are designated with an asterisk (* *p* < 0.05).

**Table 4:** Median and interquartile results of tests of all dogs studied.

Test (Units)	Ibizan Hounds	Other Breeds	Mann–Whitney *p-Value*	All Dogs
*L*. *infantum-*specific antibodies (EU)	25.96 (24.61)	24.58 (58.10)	0.8260	25.96 (35.48 EU)
SGH IgG (EU)	48.10 (26.61)	45.60 (58.10)	0.8051	47.41 (30.13)
rSP03B IgG (EU)	50.81 (50.24)	36.56 (30.77)	0.0331 *	46.75 (45.26)
LSA IFN-γ (pg/mL)	1015.59 (2122.92)	445.75 (2336.95)	0.2695	854.40 (2298.3)
SGH IFN-γ (pg/mL)	24.77 (31.36)	39.05 (106.95)	0.2379	26.75 (68.56)
rSP03B IFN-γ (pg/mL)	18.39 (37.85)	28.60 (39.35)	0.5254	19.25 (44.99)

Abbreviations: IFN-γ, interferon gamma; LSA, *L*. *infantum* soluble antigen; rSP03B, 43-kDa yellow-related recombinant protein; SGH, salivary gland homogenate. Interquartile results are expressed inside brackets. Mann–Whitney test was conducted as a two-tailed analysis. Significant *p-values* are designated with an asterisk (* *p* < 0.05).

**Table 5:** Comparison of IFN-γ LSA concentration and proportion of positive results in dogs studied based on different parameters.

		IFN-γ LSA Concentration (pg/mL)		IFN-γ LSA Frequency Result	
Variable	Number of Dogs	Median ELISA Units (IQR)	Mann–Whitney *p-Value*	Proportion Positive (Count)	Fisher’s Exact Test *p-Value*
Breed					
Ibizan Hounds	51	1015.59 (2122.92)	0.2695	40/51, 78.4%	0.4494
Other breeds	34	445.75 (2336.95)		24/34, 70.6%	
Sex					
Female	60	801.00 (2093.96)	0.4911	46/60, 76.7%	1
Male	24	1101.15 (3512.73)		18/24, 75.0%	
Age					
Young	34	388.40 (1276.59)	0.0031 **	12/34, 35.3%	<0.001 ***
Adult	50	1381.18 (2949.48)		41/50, 82.0%	
*L*. *infantum-*specific Antibodies					
Positive	30	1521.96 (2444.28)	0.0985	26/30, 86.7%	0.1133
Negative	55	731.85 (1797.05)		38/55, 69.1%	
SGH IFN-γ					
Producer	34	626.4 (2091.96)	0.6374	24/34, 70.6%	0.4494
Non-producer	51	888.25 (2341.00)		40/51, 78.4%	
rSP03B IFN-γ					
Producer	25	1172.50 (2389.61)	0.3466	21/25, 84.0%	0.2798
Non-producer	60	784.95 (2190.08)		43/60, 71.7%	
SGH IgG					
Positive	52	1255.70 (1835.57)	0.0977	40/52, 76.9%	0.7971
Negative	33	556.3 (1193.79)		24/33, 72.7%	
rSP03B IgG					
Positive	52	1098.29 (2972.81)	0.2197	39/52, 75%	1
Negative	33	556.30 (1299.65)		25/33, 75.8%	

Abbreviations: ELISA, enzyme-linked immunosorbent assay; IFN-γ, interferon gamma; LSA, *L*. *infantum* soluble antigen; rSP03B, 43-kDa yellow-related recombinant protein; SGH, salivary gland homogenate. Mann–Whitney tests were conducted as a two-tailed analysis. Significant *p-values* are designated with an asterisk (** *p* < 0.01, *** *p* < 0.001).

**Table 6:** Comparison of SGH IgG and of SP03B IgG ELISA units and proportion of positive results in dogs studied based on different parameters.

		SGH ELISA Units		SGH ELISA Result		rSP03B ELISA Units		rsp03b ELISA Result	
Variable	Number of Dogs	Median ELISA Units (IQR)	Mann–Whitney *p-Value*	Proportion Positive (Count)	Fisher’s Exact Test *p-Value*	Median-ELISA Units (IQR)	Mann–Whitney *p-Value*	Proportion Positive (Count)	Fisher’s Exact Test *p-Value*
Breed									
Ibizan Hounds	51	48.10 (26.61)	0.8051	32/51, 62.7%	0.8211	50.81 (50.24)	0.0313 *	32/51, 62.7%	0.8211
Other Breeds	34	45.60 (58.10)		20/34, 58.8%		36.56 (30.77)		20/34, 58.8%	
Sex									
Female	60	47.06 (26.44)	0.6595	37/60, 61.7%	1	46.74 (45.55)	0.7702	37/60, 61.6%	0.8084
Male	24	50.66 (56.66)		15/24, 62.5%		56.65 (47.87)		14/24, 58.3%	
Age									
Young	34	47.41 (23.27)	0.7394	22/34, 64%	0.6503	53.24 (50.26)	0.1591	25/34, 73.5%	0.1085
Adult	50	46.71 (38.12)		29/50, 58%		43.51 (43.14)		23/50, 46%	
*L*. *infantum-*Specific Antibodies									
Positive	30	54.42 (38.27)	0.0029 **	25/30, 83.3%	0.0023 **	56.65 (48.42)	0.1086	22/30, 73.3%	0.107
Negative	55	36.35 (31.93)		27/55, 49.1%		41.63 (43.47)		30/55, 54.5%	
LSA IFN-γ									
Producer	64	46.36 (28.85)	0.6948	40/64, 62.5%	0. 7971	46.76 (42.27)	0.8505	39/64, 60.9%	1
Non-Producer	21	49.88 (28.48)		12/21, 57.14%		46.75 (57.80)		13/21, 61.9%	
SGH IFN-γ									
Producer	34	39.47 (30.8)	0.163	18/34, 52.9%	0.2567	41.96 (45.04)	0.6505	20/34, 58.8%	0.8211
Non-Producer	51	49.88 (43.36)		34/51, 66.7%		46.78 (43.25)		32/51, 62.7%	
rSP03B IFN-γ									
Producer	25	36.6 (33.20)	0.1387	11/25, 44%	0.0506	39.07 (26.13)	0.3135	12/25, 48%	0.1436
Non-Producer	60	49.74 (30.49)		41/60, 68.3%		53.24 (43.97)		40/60, 66.6%	
rSP03B IgG									
Positive	52	55.65 (39.09)	<0.001 ***	43/52, 82.7%	<0.001 ***	-	-	-	-
Negative	33	24.59 (20.27)		9/33, 27.3%		-		-	
SGH IgG									
Positive	52	-	-	-	-	71.51 (53.71)	<0.001 ***	43/52, 82.7%	<0.001 ***
Negative	33	-		-		31.96 (11.54)		9/33, 27.3%	

Abbreviations: ELISA, enzyme-linked immunosorbent assay; IFN-γ, interferon gamma; LSA, *L*. *infantum* soluble antigen; rSP03B, 43-kDa yellow-related recombinant protein; SGH, salivary gland homogenate; IQR, interquartile results. Mann–Whitney tests were conducted as a two-tailed analysis. Significant *p-*values are designated with an asterisk (* *p* < 0.05, ** *p* < 0.01, *** *p* < 0.001).

**Table 7:** Comparison of IFN-γ SGH and of IFN-γ rSP03B concentrations and proportion of positive results in dogs studied based on different parameters.

		IFN-γ SGH Concentration (pg/mL)		IFN-γ SGH Result		IFN-γ rSP03B Concentration (pg/mL)		IFN-γ rSP03B Result	
Variable	Number of Dogs	Median ELISA Units (IQR) Positives (n = 34)	Mann–Whitney *p-Value*	Proportion Positive	Fisher’s Exact Test *p-Value*	Median ELISA Units (IQR) Positives (n = 25)	Mann–Whitney *p-Value*	Proportion Positive	Fisher’s Exact Test *p-Value*
Breed									
Ibizan Hounds	51	24.77 (31.36)	0.6212	18/51, 35.3%	0.3666	18.39 (37.85)	0.4342	14/51, 27.5%	0.636
Other breeds	34	39.05 (106.95)		16/34, 47.1%		28.60 (39.35)		11/34, 32.4%	
Sex									
Female	60	26.21 (32.47)	0.1913	22/60, 36.7%	0.3271	33.11 (45.57)	0.4867	16/60, 26.7%	0.4287
Male	24	77 (127.63)		12/24, 50%		18.5 (26.2)		9/24, 37.5%	
Age									
Young	34	16.29 (48.19)	0.6063	12/34, 35.3%	0.5001	27.20 (40.27)	0.9773	8/34, 23.5%	0.3404
Adult	50	29.74 (62.00)		22/50, 44.0%		19.25 (36.27)		17/50, 34%	
*L*. *infantum-*Specific Antibodies									
Positive	30	23.02 (27.73)	0.1532	11/30, 36.7%	0.8171	18.5 (37.80)	0.8931	11/30, 36.7%	0.3238
Negative	55	32.50 (96.60)		23/55, 41.8%		27.27 (37.80)		14/55, 25.5%	
LSA IFN-γ									
Producer	64	27.02 (82.10)	0.8381	24/64, 37.5%	0.4494	19.25 (45.80)	0.8027	21/64, 32.8%	0.2798
Non-Producer	21	26.44 (30.95)		10/21, 47.6%		29.47 (25.14)		4/21, 19%	
rSP03B IFN-γ									
Producer	25	32.5 (22.6)	0.3538	15/25, 60%	0.0275 *	-	-	-	-
Non-Producer	60	21.33 (91.92)		19/60, 31.7%		-		-	
SGH IFN-γ									
Producer	34	-	-	-	-	35.3 (58.88)	0.0192 *	15/34, 44.1%	0.0275 *
Non-Producer	51	-		-		2.62 (26.07)		10/51, 19.6%	
SGH IgG									
Positive	52	26.75 (70.71)	0.6212	18/52, 34.6%	0.2576	18.40 (21.38)	0.172	11/52, 21.15%	0.0359 *
Negative	33	26.70 (50.79)		16/33, 48.5%		36.08 (73.68)		14/33, 42.42%	
rSP03B IgG									
Positive	52	27.24 (53.13)	0.4783	20/52, 38.5%	0.8211	18.01 (30.14)	0.2701	12/52, 23.07%	0.1076
Negative	33	23.61 (68.49)		14/33, 42.4%		35.30 (33.10)		13/33, 39.39%	

Abbreviations: ELISA, enzyme-linked immunosorbent assay; IFN-γ, interferon gamma; LSA, *L*. *infantum* soluble antigen; rSP03B, 43-kDa yellow-related recombinant protein; SGH, salivary gland homogenate. Mann–Whitney tests were conducted as a two-tailed analysis. Significant *p-values* are designated with an asterisk (* *p* < 0.05).

## Data Availability

The data of this study are available from the corresponding author upon reasonable request.

## Abbreviations

The following abbreviations are used in this manuscript:

ELISA	enzyme-linked immunosorbent assay
IFN-γ	interferon gamma
LSA	*L. infantum* soluble antigen
rSP03B	43-kDa yellow-related recombinant protein
SGH	salivary gland homogenate
